# How is the Ethno-Socioscientific Issues (Ethno-SSI) Learning Model Javanese Culture Strengthen High School Students’ Environmental Awareness?: Observational Study

**DOI:** 10.12688/f1000research.172474.2

**Published:** 2026-05-21

**Authors:** Ahmad Khoiri, Nulngafan Nulngafan, Sigit Muryanto, Qori Agussuryani Puji Hartini, Mukhamad Fauzi, Nasokah Nasokah, Nafi Pri Nur Ratih

**Affiliations:** 1 Department of Physics Education, Universitas Sains Al-Qur'an, Wonosobo, Central Java, 56351, Indonesia; 2 Department of Informatics Management, Universitas Sains Al-Qur'an, Wonosobo, Central Java, 56351, Indonesia; 3 Department of Agrotechnology, Universitas Boyolali, Boyolali, Central Java, 57315, Indonesia; 4 Department of Elementary School Education, Universitas Sains Al-Qur'an, Wonosobo, Central Java, 56351, Indonesia

**Keywords:** Environmental Awareness, Ethnoscience, Javanese Culture, Learning Model, Strategi Socioscientific Issues (SSI)

## Abstract

**Background:**

Environmental Awareness (EA) research is part of character education that is still ignored by most students in the era of globalization. The purpose of this research is to analyze the effectiveness and contribution of the Javanese Culture Ethno-Socioscientific Issues (Ethno-SSI) Learning Model in strengthening the EA of high school students.

**Methods:**

The Design-based Research (DBR) with quantitaive and qualitative approaches. The research subjects were high school students in Wonosobo (Area 1), Boyolali (Area 2), and Temanggung Regency (Area 3) Central Java Province, Indonesia with a purposive sampling technique based on the distance of the district area to the city center (City, Middle, and Village Categories) (N = 9 high schools = 241 students). The data collection methods were standardized Fischer EA tests and questionnaires. The analysis was based on qualitative data in the form of observations of the Ethno-SSI reconstruction of Javanese culture and structured interviews, which were analyzed descriptively. Quantitative data using Statistical Product and Service Solutions (SPSS) 25.0 Analysis of Variance (ANOVA) test.

**Results:**

The results of the study show that differences in school background are not the main factor in strengthening environmental awareness, but rather based on character and science learning habits. Based on the highest average EA, the Senior high schools (SMA) Village 2 Group (SMA-V2) of 85.0068, and the lowest group is SMA village 3 (SMA-V3) of 75.5648. Based on the ANOVA test, there are significant differences and influences between each SMA category. R Squared The multiple determination value of all high school groups with EA shows an R Squared value of 0.60, meaning that the size of the independent variable can influence EA by 60%.

**Conclusions:**

The Javanese Ethno-SSI Cultural Model contributes to EA through learning designs that emphasize concern for respecting, being responsive, and preserving traditions and culture. The Ethno-SSI model is able to contribute to preserving traditions and culture by providing students with the opportunity to respond and find solutions to environmental problems.

## Introduction

Human life is inseparable from interactions with the surrounding environment. These interactions have led to the emergence of global educational problems. The use of media and learning technology as solutions to overcome these problems, ^1^ however, also leaves a negative impact on environmental damage, resulting in a decline in the quality of life. This has worsened since the development of science education, which is still separated from its natural resources. On the other hand, environmental issues remain under-researched and under-utilized in learning. ^2, 3^ In fact, the lack of integration of science learning models, the lack of teacher and student action to protect the environment, be responsible, play an active role, explore environmental issues and local wisdom, and the inability to manage the environment wisely. ^4^

This challenge is a key issue for analyzing through Environmental Awareness (EA) among high school students in Central Java. Central Java Province, consisting of 35 regencies and cities, possesses a wealth of local wisdom that can be used as a learning resource. The theme “Javanese Culture” is expected to empower students to understand, feel ownership of, and assume responsibility as a generation with environmental character.

The importance of strengthening EA stems from the increasing globalization that is eroding community culture ^5, 6^ and the need to prepare competitive graduates by improving student learning performance, ^7^ while still upholding cultural traditions.

The results of a random survey of 15 teachers and 256 high school students in Wonosobo Regency, Central Java Province, Indonesia (the area of origin of the lead researcher) in January 2025 observed that schools had progress of 75% in environmental policies, 62% in environmentally based curriculum, 56% in developing environmental activities, 55% in managing environmentally friendly supporting facilities, and the most concerning result was that 80% of students expressed concern for the environment, but had not been able to contribute to overcoming its damage. The survey results were strengthened by research ^8^ that the low implementation of environmentally based learning resulted in a lack of positive character in students. The solution so that students are able to acquire knowledge and understanding of environmental problems that will change their attitudes and awareness of their social life, ^9, 10^ requires the design of effective and contextual learning models.

Based on research experience, education based on local wisdom and cultural traditions requires strategies, methods, approaches, and learning models to empower students’ skills and positive attitudes. ^11– 17^ Specific learning strategies that involve morals and present social issues are called Socioscientific Issues (SSI) Strategies ^18, 19^ are one solution for constructing a learning model based on local wisdom, ethnoscience, and character.

EA analysis through model design is aimed not only at developing an attitude of caring and preserving the environment, but also providing real contributions through policy recommendations and sustainable environmental education programs. ^20– 22^ The research problem formulation analyzes how the effectiveness and contribution of the Ethno-SSI Learning Model “Javanese Culture” in strengthening the EA of high school students in Central Java. The problem-solving approach regarding the Characteristics of the Ethno-SSI Model, which carries the theme “Javanese Culture” as a solution to the learning model for social issues based on local wisdom as a form of appreciation for the local wealth owned by Central Java Province, and is very possible to be adopted in other regions.

The characteristics of the model have components of meaningful learning syntax, environmentally oriented social systems, principles of student and teacher reactions, and the impact of strengthening EA in preserving cultural heritage in Central Java Province. The importance of mapping students’ EA profiles as one of the indicators of achieving the community development index at high school age. The low environmental awareness of high school students is a particular concern regarding the challenges in preparing Indonesia’s golden generation of 2045. The EA indicator is a benchmark for the success of a generation that cares and is not apathetic towards environmental responses through information on the effectiveness and extent of the model’s contribution to producing recommendations for local wisdom-based education policies.

Based on the research problem, the purpose of this study is to analyze the effectiveness and contribution of the Javanese Cultural Ethno-SSI Learning Model in strengthening the EA of high school students. Javanese culture is found in Central Java Province, Indonesia.

## Methods

**Figure 1.**
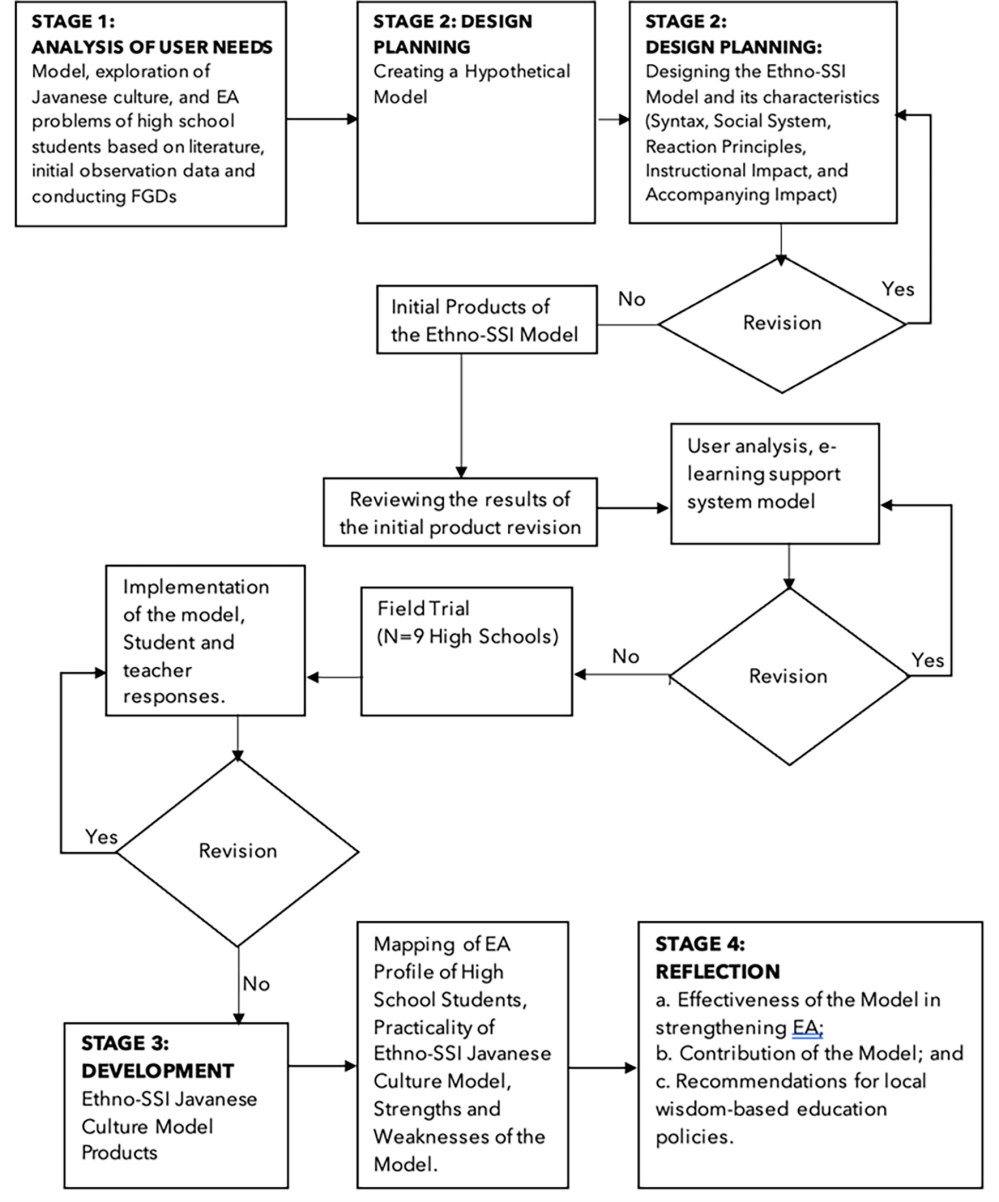
Design based Research (DBR) flowchart to provide a procedure consisting of four stages, namely: Analysis of user needs, product design planning for the Ethno-SSI Javanese Culture Model, followed by field trials and model development and carrying out reflection on research results.

The research procedure, Design-Based Research (DBR) ^23^ ( Figure 1), was used to understand the objectives of the Ethno-SSI Javanese Culture model, which has an instructional impact on high school students’ EA. DBR was adapted to produce a model product consisting of four steps: needs analysis, design planning, development, and reflection of results. In the needs analysis step, the researcher identified the needs regarding the ethno-SSI Javanese Culture model. Based on the needs analysis, the initial model design was developed in the planning, design, and development stages. Finally, the successful use of the model was evaluated in the reflection stage to determine the model’s effectiveness and recommend policies.

### Research subjects

The study subjects were 9 high schools in 3 regencies in Central Java Province, totaling 241 students.

The sample was determined based on the purposive sampling technique, meeting the research criteria, that is, the family background of students who lived in the central area of the city who all had similar attributes in terms of cultural and environmental recognition.

Purposive sampling was used based on the distance between the schools and the city or regency center: Wonosobo Regency (Area 1), Boyolali Regency (Area 2), and Temanggung Regency (Area 3), divided into three categories (city, middle, and village). The selection of the district area as the research location was based on the fulfillment of Javanese culture that was relevant to the research theme. Sample size based on the number of classes taken randomly. This selection was based on cultural backgrounds and emerging ethno-SSI issues within the community. The study sample consisted of 9 high schools: Area 1: SMA-C1 (city), SMA-M1 (middle), SMA-V1 (village); Area 2: SMA-C2 (city), SMA-M2 (middle), SMA-V2 (village); Area 3: SMA-C3 (city), SMA-M3 (middle), SMA-V3 (village).

SMA-C1 is the first school located in the city at a distance of 1-5 km from the district center of Wonosobo Regency.

SMA-M1 is the first school located in Madya at a distance of 6-15 km from the district center of Wonosobo Regency.

SMA-V1 is the first school located in a village at a distance of more than 15 km from the district center of Wonosobo Regency.

For schools in other districts, the same thing applies. ^23^

The inclusion requirements of this study were as follows.

1) Male and female students who are actively registered as students at 9 high schools in 3 regencies in Central Java Province Indonesia.
2) Willing to become informants.
3) Physically and mentally healthy.
4) Students’ responses are not influenced by the opinions of teachers, friends, guardians of students or others.
5) Students in class 10-12 only.

Figure 1 at the development stage (part of DBR design), trials are carried out using experimental methods with a pre-test/post-test control group design (see Table 1). ^23^

**Table 1.** Research design.

Group	O	Treatment	O
Experiment Group	O1	Ethno-SSI Model	O2
Control Group	O3	Ethnoscience Model	O4

### Data collection methods

The Fischer standardized Environmental Awareness (EA) test consisted of 8 essay questions, and the EA questionnaire consisted of 36 items. Observations of the Ethno-SSI reconstruction of Javanese culture and structured interviews were conducted with cultural experts, teachers, and students. The data collection instruments were structured based on the fulfillment of the indicators adapted from Fischer: Care (EA1), Curiosity (EA2), Criticality (EA3), Dependability (EA4), Responsibility (EA5), and Local Wisdom (EA6). ^23^

Data collection was carried out over a period of one semester (May to October 2025). EA Instruments and Measurement Scales at the link: https://forms.gle/iRr4LvPzQ44MNyMT6 (Wonosobo Regency), https://forms.gle/iNbWYMjnfQcsWYjw8 (Boyolali Regency), https://forms.gle/udCz7SgV5PyfRKf27 (Temanggung Regency).

### Data analysis techniques

The analysis was based on qualitative data in the form of observations of the Ethno-SSI reconstruction of Javanese culture and structured interviews, which were analyzed descriptively. Quantitative data, in the form of EA test results and EA questionnaires, were analyzed. Prerequisite tests for normality and homogeneity were followed by t-tests and Analysis of Variance (ANOVA) to test the effectiveness of the Ethno-SSI model in strengthening students’ EA. Data interpretation was used to make recommendations for local wisdom-based education policies.

To address data bias, researchers selected samples from various school backgrounds to anticipate similar responses. Furthermore, the environmental awareness test was descriptive, allowing for differences in each response. However, researchers had already identified the variable indicators used in the study.

### Ethical considerations

The Higher Education, Science, and Technology Indonesia Republic for the Regular Fundamental Research on May 28, 2025 (letter number 127/C3/DT.05.00/PL/2025 and 027/LL6/PL/AL.04/2025) is the institution that has approved and provided funding for this research.

This research was also approved by the Institute for Research, Publishing, and Community Service of the Universitas Sains Al-Quran (UNSIQ), Wonosobo, Central Java, Indonesia granted permission for this research on July 1, 2025 (letter number 120/LP3M-UNSIQ/VII/2025).

Written informed consent to participate was obtained from all participants *(students*, teachers, and the principal). If teacher consent was not obtained, the student was not permitted to participate. Respondents provided consent without coercion from anyone. All data collected will be kept confidential to protect the rights and privacy of respondents.

### Consent to participation

Consent to participate in the study was obtained based on the Statement from the Committee on the Use of Human Subjects as Experimental Subjects (COUHES) at https://couhes.mit.edu/informed-consent .

This statement provides detailed and clear information regarding:

1) Title and theme of the study
2) Treatment to be applied to the subjects
3) Benefits of participating as a research subject
4) Potential dangers
5) Understanding of Research Procedures
6) Approval of research site permits
7) Rights to security and privacy and research procedures. The opportunity to ask questions regarding anything related to the study.

Because the participants have voluntarily agreed to be research subjects, they are fully aware and without coercion. This statement is made truthfully and without pressure from any party.

Consent is based on https://www.csusm.edu/research/compliance/irb/consent.html about Informed Consent and Assent Process and Forms (ICAPF). This consent is based on information that must still be obtained from the subject’s parents or guardians, because this study is aimed at high school students aged 15-17 years.

Parental consent was obtained by distributing a research consent form with the seven criteria above. If informed consent was approved, the form was signed by the student’s parents.

## Results

### Characteristics of the Ethno-SSI learning model “Javanese Culture”

The characteristics of the Ethno-SSI Model, which carries the theme “Javanese Culture,” are a solution to social issues based on local wisdom, a form of appreciation for the local richness of Central Java Province, and have the potential for adoption in other regions. The model’s characteristics include meaningful learning syntax, an environmentally oriented social system, the principle of student and teacher responsiveness, and the impact of strengthening EA in preserving cultural heritage in Central Java Province.

The importance of the Javanese Ethno-SSI Cultural Model in mapping students’ EA profiles as an indicator of the achievement of the community development index at high school age, and supporting Indonesia’s Asta Cita. The low environmental awareness of high school students is a particular concern, challenging the preparation of Indonesia’s golden generation for 2045. The results of the Ethno-SSI reconstruction in three districts include: the Dreadlock Traditional Ritual, the Tiban Market Tradition, and the Wiwit Mbako Traditional Ritual.

### Dreadlock ritual tradition

Social issues that develop in the Dieng Plateau are located in two districts, namely Wonosobo and Banjarnegara, Central Java Province, which not only holds beauty, but also the mystery of the dreadlocked boy who appears every time. The dreadlocked boy is said to be the incarnation of Kyai Kolodete and Nini Roro Ronce. The dreadlocks appear with a sign that the child is experiencing a fever, so that the child’s dreadlocks can be cut, a Ruwatan must be performed, and a hair-cutting ritual must be followed. The cutting of the child’s dreadlocks is carried out using water taken from Sendang Sedayu, located in the corner of the Dharmasala complex. After leaving Dharmasala, the child is carried by his parents into the Arjuna Temple. The temple complex has prepared a place for the dreadlock-cutting procession. The procession is led by the Dieng traditional leader, Mbah Sumanto, before having their hair cut, the children have requested something that must be realized during the haircut, and are informed of the public about each child’s request.

The ceremony begins with the chanting of the Dandanggula macapat song, followed by the cutting of the child’s dreadlocks. The traditional elder, Mbah Sumanto, initiates the shaving. The cut hair is placed in a jar. The hair is then floated down a river that flows to the Indian Ocean. Telaga Warna is typically the location for the floating.

Dreadlocks grow in children aged one to five years. The initial appearance of dreadlocks is usually accompanied by symptoms such as a high fever, and some even experience convulsions. It is said that the child can recover from the fever after the dreadlocks have finished growing. An ethnoscientific explanation and its integration with physics concepts are presented in Table 2.

**Table 2.** Science reconstruction in dreadlocks tradition.

Indigenious Science	Natural Science	Awareness Indicator
*The Dreadlocked Boy is said to be the incarnation of Kyai Kolodete and Nini Roro Ronce.*	There isn’t any	
It starts with a fever or high temperature.	Temperature measuring instrument, thermometer	Curiosity (EA2), Criticality (EA3)
In order for a child’s dreadlocks to be cut, a Ruwatan must be performed and a hair cutting ritual must be followed.	There isn’t any	
Cutting of Children’s Dreadlocks is done by using water taken from Sendang Sedayu. It is located in the corner of the Dharmasala complex.	Energy Transfer	Dependability (EA4), Responsibility (EA5)
The procession was led by the Dieng traditional elders. Before their hair was cut, the children had asked for something that had to be realized during the hair cutting.	There isn’t any	
The procession begins with the singing of the Dandanggula macapat song, the child’s dreadlocks are cut.	There isn’t any	
The hair that has been cut is put into a jar. The hair that has been cut is thrown into a river that flows to the Indian Ocean.	Energy Transfer	Local Wisdom (EA6)

The Ruwatan Rambut Gimbal tradition is closely linked to local beliefs and the mythology of the Dieng people. ^24, 25^ The cutting process, or ruwatan, is carried out through a sacred ceremony involving traditional leaders, families, and the surrounding community. This tradition reflects local wisdom that combines elements of spirituality, respect for nature, and traditional social structures.

The Ruwatan Rambut Gimbal ceremony in Wonosobo not only contains spiritual and social values but is also imbued with valuable ecological messages to foster environmental awareness among the younger generation. In each stage, this tradition demonstrates how local communities respect nature, maintain its balance, and use natural resources wisely and measurably. This aligns with modern scientific principles of efficiency, conservation, and sustainable environmental management.

One concrete example of environmental awareness is the use of water from seven natural springs as a means of purification during the ritual. Water is not taken in large quantities haphazardly, but is measured traditionally using local, environmentally friendly containers, such as clay jugs. This is an example of the application of volume measurement in a simple yet sustainable manner. In the context of physics, this teaches the importance of efficient and moderate volume measurement, while also teaching students about water conservation.

The offerings, such as flowers, leaves, and food, used in the ceremony also demonstrate the principle of utilizing local, biodegradable resources and not producing inorganic waste. There is no plastic, Styrofoam, or other synthetic materials. The community uses natural measuring instruments, such as banana leaves as bases, bamboo segments as water containers, or handfuls of food. This demonstrates how local culture has long embraced concepts now recognized in modern science as environmentally friendly and ecologically based.

### *Tiban* market tradition

One of the social issues developing in Boyolali Regency, Central Java Province, is the Tiban Market, a traditional market selling food and souvenirs typical of the Selo people. This market is held when groups of guests visit and purchase village tourism packages. The tourism experience offered is a combination of typical Selo snacks and cultural arts attractions ( Table 3). This is complemented by the beautiful views of Mount Merapi. The uniqueness of this market is not only the commodities sold; transactions also use tokens in the form of coins. These coins are made from coconut shells and are worth Rp 2,000. To entertain visitors who shop and eat snacks, children from the studio perform traditional dances and warm the village atmosphere. All vendors are free of charge to the Pokdarwis (tourism group). Profit sharing occurs when sellers exchange the coconut shell coins they collect from buyers with the Pokdarwis management.

**Table 3.** Science reconstruction “tiban market tradition”.

Indigenious Science	Natural Science	Awareness Indicator
Tiban Market is a people’s market that sells food and souvenirs typical of Selo residents.	Chemicals in food at the tiban market	
The uniqueness of this market is not only the commodities being sold, but the buying and selling transactions also use tokens in the form of coins.	Utilization of natural materials in coconut shells	Care (EA1)
Profit sharing occurs when sellers exchange the coconut shell coins they collect from buyers back to the Pokdarwis managers.	Bioenergy in coconut shell coins can be made into briquettes	Criticality (EA3), Responsibility (EA5)

The use of coconut shell coins as evidence of waste utilization for financial transactions at the tiban market demonstrates community awareness of coconut waste utilization. The EA indicator is a form of responsibility, where students can respond to the problem of accumulating waste or waste that has a market value in the community. One example is the creation of briquettes from coconut shell waste. Briquettes are organic biomass that can produce specific energy as fuel. Energy transformation can be studied through scientific concepts, so students indirectly learn about cultural traditions and science simultaneously.

### *Wiwit Mbako* ritual tradition

The social issue that is developing in the Temanggung Regency community is “Wiwit mbako,” which is a traditional community ritual that marks the start of the tobacco harvest. This ritual is an expression of gratitude for the expected good and blessed harvest, while preserving local wisdom ( Table 4). There are cultural values in the implementation of the Wiwit mbako tradition, namely spiritual values, vital values, and formal values. These values can be identified from the elements of the implementation of the Wiwit tobacco tradition, namely umbarape, determining the day of implementation and the rituals carried out. The implementation of the Wiwit tobacco tradition has gone through three stages of cultural strategy, namely the mystical stage, the ontological stage, and the functional stage. The mythical realm of thought is seen in the belief in Ki Ageng Makukuhan and Dewi Sri, the ontological realm of thought is reflected in the change in implementation from initially individual to group, and the functional realm of thought is seen in its function as cultural tourism. Profit sharing occurs when sellers exchange the coconut shell coins they collect from buyers with the Pokdarwis manager, as presented in Table 4.

**Table 4.** Science reconstruction in ritual tradition “wiwit mbako”.

Indigenious Science	Natural Science	Awareness Indicator
Wiwit mbako is a form of gratitude to God for the tobacco harvest which is expected to be of good quality and profitable for farmers.	There isn’t any	
This ritual is part of Temanggung’s cultural heritage that needs to be preserved.	There isn’t any	Care (EA1)
Joint Prayer: The wiwit mbako procession usually begins with a joint prayer in the tobacco fields, asking for a smooth harvest and satisfactory results.	There isn’t any	
The first picking of tobacco leaves by community leaders or related officials using tools.	Tobacco picking tool using a simple machine type	Criticality (EA3), Dependability (EA4)
After the picking, usually a communal meal is held (kembul bujono) as a form of togetherness.	There isn’t any	
Wiwit Mbako also strengthens the spirit of cooperation and togetherness among tobacco farming communities.	There isn’t any	
With the presence of wiwit mbako, it is hoped that the quality of Temanggung tobacco will improve and the price will also increase.	Lighting and humidity factors in tobacco plant growth	Curiosity (EA2), Criticality (EA3)
The wiwitan tradition has now undergone changes related to the changing times (modernization). These changes are seen in the determination of the day, the implementation process and changes in the uborampe (materials and tools) in the wiwitan tradition.	The determination of the change in day is based on Global Warming; uncertain climate change is one of the factors that causes changes in time during the tobacco planting and harvesting process	Dependability (EA4), Local Wisdom (EA6)

Based on in-depth interviews, it was revealed that in the past, people always used auspicious days, established by their predecessors, to determine the date for the Wiwitan tradition, which is a large event according to the Javanese calendar. However, those who still practice the Wiwitan tradition no longer adhere to the days considered auspicious by their predecessors. This is due to a shift in the community’s mindset, which considers all days auspicious. Furthermore, the Mranggen Tengah Village community held the Sadranan ceremony, which was not held on Wednesday *Pahing* but on Friday *Legi.*

The EA indicator focuses on students’ understanding of natural conditions during tobacco planting and harvesting. Students can learn about weather changes due to global warming. Students can learn about the appropriate weather conditions for planting tobacco for a bountiful harvest. Furthermore, the tools used to harvest tobacco utilize simple machines, such as sickles, lawn mowers, and other tools. Students can understand the types of simple machines used in tobacco planting and harvesting.

### Statistical test results

The participant demographics consisted of 9 high schools in 3 different districts. The total number of students was 241 (SMA-C1, N = 33; SMA-M1, N = 27; SMA-V1, N = 30; SMA-C2, N = 23; SMA-M2, N = 25; SMA-V2, N = 22; SMA-C3, N = 27; SMA-M3, N = 29; and SMA-V3, N = 25). The number of male students was 102, and female students was 139. Based on the school background, there are 83 students in the city category, 81 students in the middle category of high school and 77 students in the village category of high school. the entire data in the research trial to respond to the EA instrument. Next, after the data is collected, statistical test analysis is carried out.

Normality and homogeneity test

The normality test for Environmental Awareness (EA) of students in each high school group in Area 1: SMA-C1 (city), SMA-M1 (medium), SMA-V1 (village). Area 2: SMA-C2 (city), SMA-M2 (medium), SMA-V2 (village). Area 3: SMA-C3 (city), SMA-M3 (medium), SMA-V3 (village) using the Kolmogorov test with Lilliefors correction ^26^ is presented in Table 5. The normality test for EA for each high school group is as follows ( Table 5):

1. High School Group 1 City (SMA-C1) L-value is 0.114 with p-value 0.200 > 0.05, then accept H0, which means that the EA of Students in High School Group 1 City (SMA-C1) is normally distributed.
2. High School Group 1 Medium (SMA-M1) L-value is 0.164 with p-value 0.059 > 0.05, then accept H0, which means that the EA of Students in High School Group 1 Medium (SMA-M1) is normally distributed.
3. High School Group 1 Village (SMA-V1) L-value is 0.102 with p-value 0.200 > 0.05, then accept H0, which means that the EA of Students in High School Group 1 Village (SMA-V1) is normally distributed.

**Table 5.** Tests of environmental awareness normality.

SMA Group	Kolmogorov-Smirnov ^a^			Shapiro-Wilk		
	Statistic	df	sig	Statistic	df	sig
SMA-C1	.114	33	.200 *	.965	33	.363
SMA-M1	.164	27	.059	.946	27	.169
SMA-V1	.102	30	.200 *	.965	30	.419
SMA-C2	.181	23	.048	.931	23	.114
SMA-M2	.146	25	.181	.956	25	.332
SMA-V2	.156	22	.178	.931	22	.126
SMA-C3	.203	27	.006	.789	27	.000
SMA-M3	.158	29	.061	.938	29	.090
SMA-V3	.296	25	.000	.801	25	.000

* This is a lower bound of the true significance.

^a^ Lilliefors Significance Correction.

Because most high school groups showed normally distributed Student EA values, it is highly likely that the residuals are normally distributed, thus meeting the assumption of normality. If this is not met, bootstrapping is performed to ensure consistent parameter estimates despite violations of normality.

The homogeneity of variance for Student EA between groups was tested using the Levene test, as presented in Table 6.

**Table 6.** Test of enviromental awareness homogeneity of variance.

	Levene			
	Statistic	df1	df2	sig
Based on Mean	1.361	8	232	.215
Based on Median	1.074	8	232	.382
Based on Median and with adjusted df	1.074	8	154.814	.384
Based on trimmed mean	1.259	8	232	.266

The homogeneity test for Student EA shows a p-value of 0.215 > 0.05, thus accepting H0, which means that the variances for Student EA between groups are not significantly different, also known as homogeneous. Therefore, the assumption of homogeneity is met. If this is not met, the post hoc test will use the Games-Howell test. Alternatively, non-parametric tests such as Kruskal-Wallis and Mann-Whitney will be used. The normality test for Student EA in each group will be tested using the Normal QQ and Detrended QQ PLOT graphs:

1. Normal QQ: If the plot follows the diagonal line, it is normally distributed. Otherwise, it is not normally distributed.
2. Detrended QQ: If the plot is evenly distributed above and below the 0 axis, it is normally distributed. Otherwise, it is not normally distributed.

### Analysis of Variance (ANOVA) test

There are 9 high school groups, based on Table 7. The descriptive test of student EA per high school group shows that the highest mean student EA is in the Village 2 High School Group (SMA-V2), at 85.0068. Meanwhile, the lowest is in the Village 3 High School Group (SMA-V3), at 75.5648. Furthermore, to determine whether the difference in student EA is significant, an ANOVA test is used, as presented in Table 8.

**Table 7.** Between-subjects factors.

		Value label	N
SMA Group	1.00	SMA-C1	33
	2.00	SMA-M1	27
	3.00	SMA-V1	30
	4.00	SMA-C2	23
	5.00	SMA-M2	25
	6.00	SMA-V2	22
	7.00	SMA-C3	27
	8.00	SMA-M3	29
	9.00	SMA-V3	25

**Table 8.** Descriptive statistics.

SMA group	Dependent Variable: Environmental Awareness		
	Mean	Std. Deviation	N
SMA-C1	82.7870	8.67558	33
SMA-M1	76.1785	6.51623	27
SMA-V1	79.3680	5.85738	30
SMA-C2	79.9183	7.26995	23
SMA-M2	81.1084	9.86715	25
SMA-V2	85.0068	8.51696	22
SMA-C3	79.6104	12.8809	27
SMA-M3	83.3286	9.73917	29
SMA-V3	75.5648	12.4478	25
Total	80.3359	9.62930	241

Based on the student EA, using a one-way ANOVA test, the effect of the high school group on student EA is 0.004 < 0.05, thus accepting H1, meaning the high school group significantly influences student EA, as presented in Table 9. Furthermore, the student EA for the one-way ANOVA test is presented in Table 10, showing the output of the one-way ANOVA analysis. Several studies need to be conducted to determine whether there is a significant influence or difference between the high school groups on student EA. The following is an explanation of each output of the One-Way ANOVA analysis, including:

1. Corrected Model: The effect of all independent variables (High School Group) together on the dependent variable (Student EA). If Sig.) <0.05 (Alpha) = Significant. Table 10 shows Student EA. Sig. 0.000 < 0.05 means the model is valid and the data is significant.
2. Intercept: The value of the change in the dependent variable (EA) without the presence of the independent variable (Ethno-SSI Javanese Culture Model), meaning that without the influence of the independent variable, the dependent variable can change its value. If (Sig.) <0.05 (Alpha) = Significant. A value shown in the table of Sig. 0.000 < 0.05 means the Intercept is Significant.
3. High School Group: The effect of High School Group on the value of the Student EA measurement matrix in the model. If (Sig.) <0.05 (Alpha) = Significant. Table 10 shows the Sig. Value. High School Group 0.004 < 0.05 means that the high school group has a significant effect on student EA.
4. Error: Model error value; the smaller the model, the better the model.
5. R Squared: The multiple determination value of all independent variables with the dependent variable. Table 10 shows an R-squared value of 0.60, meaning that the size of the independent variable can influence the dependent variable by 60%.

**Table 9.** ANOVA.

	Sum of Squares	df	Mean Square	F	Sig.
Between Groups	2034.97	8	254.371	2.919	.004
Within Groups	20218.67	232	87.149		
Total	22253.64	240			

**Table 10.** Tests of between-subjects effects.

Source	Type III Sum of Squares	df	Mean Square	F	Sig.
Corrected Model	2034.971 ^a^	8	254.371	2.919	.004
Intercept	1531815.602	1	1531815.602	17576.884	.000
SMA group	2034.971	8	254.371	2.919	.004
Error	20218.670	232	87.149		
Total	1577631.225	241			
Corrected Total	2034.971 ^a^	8	254.371	2.919	.004

^a^ R Squared = ,91 (Adjusted R Squared = ,60).

The parameter estimate (B) for the SMA Group of the ANOVA equation model is presented in Table 11. Where Ŷ is the predicted student EA. For example, for the SMA Group in the SMA Group, the predicted Student EA is Ŷ1 = 75,565 + 7,222X = 82.787. The 95% confidence interval shows that the parameter estimate for the SMA Group of City 1 (SMA-C1) is between 2,345 and 12,099, where this parameter is significant because the p value is 0.004 < 0.05, or accept H1. Meanwhile, the 95% confidence interval for [SMA Group = 9.00] is not calculated because in this ANOVA equation, the reference used is SMA Group 9 ( Table 12). The abundance of the Tukey test above shows that:

1. The difference in the high school group between SMA Kota 1 (SMA-C1) and SMA Madya 1 (SMA-M1) is 6.60 with a p-value of 0.144 > 0.05, thus accepting H0, meaning there is no significant difference between the two.
2. The difference in the high school group between SMA Kota 1 (SMA-C1) and SMA Desa 1 (SMA-V1) is 3.42 with a p-value of 0.876 > 0.05, thus accepting H0, meaning there is no significant difference between the two.
3. The difference in the high school group between SMA Kota 1 (SMA-C1) and SMA Kota 2 (SMA-C2) is 2.87 with a p-value of 0.969 > 0.05, thus accepting H0, meaning there is no significant difference between the two.
4. Those with a p-value < 0.05 are significantly different ( Table 13).

**Table 11.** Parameter estimates of environmental awareness.

Parameter	B	Std. Error	t	Sig.	95% Confidence Interval	
					Lower Bound	Upper Bound
Intercept	75.565	1.867	40.472	.000	71.886	79.243
[SMAGroup = 1,00]	7.222	2.475	2.918	.004	2.345	12.099
[SMAGroup = 2,00]	.614	2.591	.237	.813	-4.491	5.719
[SMAGroup = 3,00]	3.803	2.528	1.504	.134	-1.178	8.784
[SMAGroup = 4,00]	4.353	2.697	1.614	.108	-.961	9.668
[SMAGroup = 5,00]	5.544	2.640	2.099	.037	.341	10.746
[SMAGroup = 6,00]	9.442	2.729	3.460	.001	4.065	14.819
[SMAGroup = 7,00]	4.046	2.591	1.561	.120	-1.059	9.151
[SMAGroup = 8,00]	7.764	2.548	3.047	.003	2.744	12.784
[SMAGroup = 9,00]	0 ^a^	.	.	.	.	.

^a^ This parameter is set to zero because it is redundant.

**Table 12.** Post Hoc Tests SMA group.

Multiple Comparisons						
Dependent Variable: Environmental Awareness						
Tukey HSD						
(I) SMA Group	(J) SMA Group	Mean Difference (I-J)	Std. Error	Sig.	95% Confidence Interval	
					Lower Bound	Upper Bound
SMA-C1	SMA-M1	6.6085	2.42253	.144	-.9773	14.1942
	SMA-V1	3.4190	2.35497	.876	-3.9552	10.7932
	SMA-C2	2.8687	2.53574	.969	-5.0715	10.8090
	SMA-M2	1.6786	2.47525	.999	-6.0723	9.4294
	SMA-V2	-2.2198	2.56948	.995	-10.2657	5.8261
	SMA-C3	3.1766	2.42253	.927	-4.4091	10.7623
	SMA-M3	-.5417	2.37614	1.000	-7.9821	6.8988
	SMA-V3	7.2222	2.47525	.090	-.5287	14.9730
SMA-M1	SMA-C1	-6.6085	2.42253	.144	-14.1942	.9773
	SMA-V1	-3.1895	2.47644	.934	-10.9440	4.5651
	SMA-C2	-3.7397	2.64894	.892	-12.0344	4.5550
	SMA-M2	-4.9299	2.59109	.613	-13.0434	3.1837
	SMA-V2	-8.8283 ^*^	2.68125	.031	-17.2242	-.4324
	SMA-C3	-3.4319	2.54077	.915	-11.3878	4.5241
	SMA-M3	-7.1501	2.49658	.103	-14.9677	.6675
	SMA-V3	.6137	2.59109	1.000	-7.4998	8.7273
SMA-V1	SMA-C1	-3.4190	2.35497	.876	-10.7932	3.9552
	SMA-M1	3.1895	2.47644	.934	-4.5651	10.9440
	SMA-C2	-.5503	2.58729	1.000	-8.6519	7.5514
	SMA-M2	-1.7404	2.52803	.999	-9.6565	6.1757
	SMA-V2	-5.6388	2.62037	.441	-13.8441	2.5664
	SMA-C3	-.2424	2.47644	1.000	-7.9969	7.5122
	SMA-M3	-3.9606	2.43108	.788	-11.5731	3.6519
	SMA-V3	3.8032	2.52803	.853	-4.1129	11.7193
SMA-C2	SMA-C1	-2.8687	2.53574	.969	-10.8090	5.0715
	SMA-M1	3.7397	2.64894	.892	-4.5550	12.0344
	SMA-V1	.5503	2.58729	1.000	-7.5514	8.6519
	SMA-M2	-1.1901	2.69724	1.000	-9.6361	7.2558
	SMA-V2	-5.0886	2.78396	.664	-13.8061	3.6290
	SMA-C3	.3079	2.64894	1.000	-7.9868	8.6026
	SMA-M3	-3.4104	2.60658	.928	-11.5724	4.7517
	SMA-V3	4.3535	2.69724	.796	-4.0925	12.7994
SMA-M2	SMA-C1	-1.6786	2.47525	.999	-9.4294	6.0723
	SMA-M1	4.9299	2.59109	.613	-3.1837	13.0434
	SMA-V1	1.7404	2.52803	.999	-6.1757	9.6565
	SMA-C2	1.1901	2.69724	1.000	-7.2558	9.6361
	SMA-V2	-3.8984	2.72898	.886	-12.4438	4.6469
	SMA-C3	1.4980	2.59109	1.000	-6.6155	9.6116
	SMA-M3	-2.2202	2.54777	.994	-10.1981	5.7577
	SMA-V3	5.5436	2.64045	.476	-2.7245	13.8117
SMA-V2	SMA-C1	2.2198	2.56948	.995	-5.8261	10.2657
	SMA-M1	8.8283 ^*^	2.68125	.031	.4324	17.2242
	SMA-V1	5.6388	2.62037	.441	-2.5664	13.8441
	SMA-C2	5.0886	2.78396	.664	-3.6290	13.8061
	SMA-M2	3.8984	2.72898	.886	-4.6469	12.4438
	SMA-C3	5.3964	2.68125	.536	-2.9994	13.7923
	SMA-M3	1.6782	2.63941	.999	-6.5867	9.9431
	SMA-V3	9.4420 ^*^	2.72898	.018	.8967	17.9874
SMA-C3	SMA-C1	-3.1766	2.42253	.927	-10.7623	4.4091
	SMA-M1	3.4319	2.54077	.915	-4.5241	11.3878
	SMA-V1	.2424	2.47644	1.000	-7.5122	7.9969
	SMA-C2	-.3079	2.64894	1.000	-8.6026	7.9868
	SMA-M2	-1.4980	2.59109	1.000	-9.6116	6.6155
	SMA-V2	-5.3964	2.68125	.536	-13.7923	2.9994
	SMA-M3	-3.7183	2.49658	.860	-11.5359	4.0994
	SMA-V3	4.0456	2.59109	.825	-4.0680	12.1591
SMA-M3	SMA-C1	.5417	2.37614	1.000	-6.8988	7.9821
	SMA-M1	7.1501	2.49658	.103	-.6675	14.9677
	SMA-V1	3.9606	2.43108	.788	-3.6519	11.5731
	SMA-C2	3.4104	2.60658	.928	-4.7517	11.5724
	SMA-M2	2.2202	2.54777	.994	-5.7577	10.1981
	SMA-V2	-1.6782	2.63941	.999	-9.9431	6.5867
	SMA-C3	3.7183	2.49658	.860	-4.0994	11.5359
	SMA-V3	7.7638	2.54777	.063	-.2141	15.7417
SMA-V3	SMA-C1	-7.2222	2.47525	.090	-14.9730	.5287
	SMA-M1	-.6137	2.59109	1.000	-8.7273	7.4998
	SMA-V1	-3.8032	2.52803	.853	-11.7193	4.1129
	SMA-C2	-4.3535	2.69724	.796	-12.7994	4.0925
	SMA-M2	-5.5436	2.64045	.476	-13.8117	2.7245
	SMA-V2	-9.4420 ^*^	2.72898	.018	-17.9874	-.8967
	SMA-C3	-4.0456	2.59109	.825	-12.1591	4.0680
	SMA-M3	-7.7638	2.54777	.063	-15.7417	.2141

Based on observed means. The error term is Mean Square (Error) = 87,149.

^*^ The mean difference is significant at the ,05 level.

**Table 13.** Tukey HSD ^a^ ^,^ ^b^ ^,^ ^c^ of environmental awareness.

SMA Group		Subset	
	N	1	2
SMA-C1	25	75.5648	
SMA-M1	27	76.1785	
SMA-V1	30	79.3680	79.3680
SMA-C2	27	79.6104	79.6104
SMA-M2	23	79.9183	79.9183
SMA-V2	25	81.1084	81.1084
SMA-C3	33	82.7870	82.7870
SMA-M3	29	83.3286	83.3286
SMA-V3	22		85.0068
Sig.		.068	.413

Means for groups in homogeneous subsets are displayed.

Based on observed means.

The error term is Mean Square (Error) = 87,149.

^a^ Uses Harmonic Mean Sample Size = 26,383.

^b^ The group sizes are unequal. The harmonic mean of the group sizes is used. Type I error levels are not guaranteed.

^c^ Alpha = ,05.

**Figure 2.**
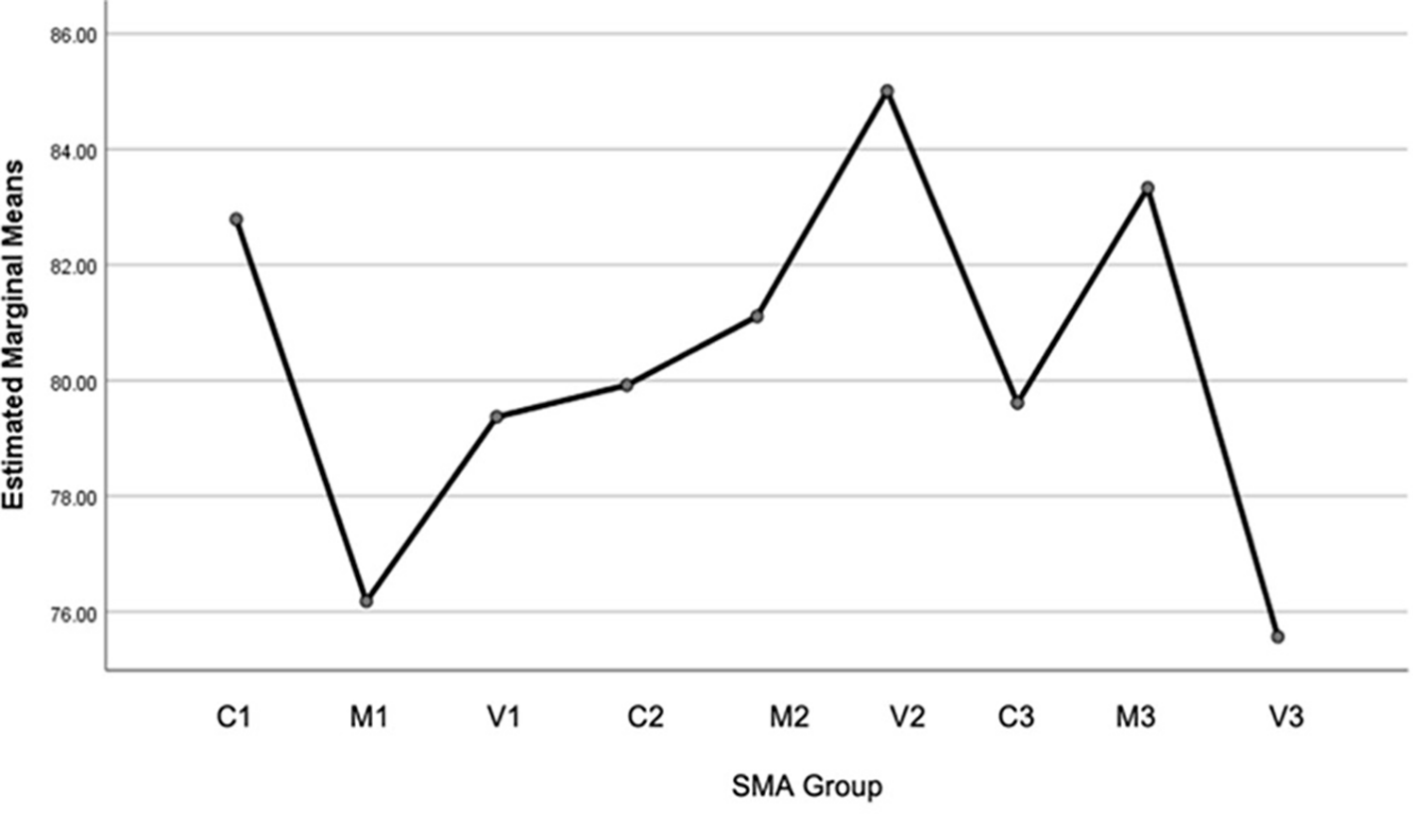
Data Profile plots of environmental awareness for 9 schools based on comparison of regional categories (City, Middle, Village) spread across 3 districts (Wonosobo, Boyolali and Temanggung), Central Java, Indonesia.

Furthermore, the average EA profile plots are shown in the values in the same subset; there is no difference, while the values in different subsets mean there is a difference. Figure 2 shows the difference in the average EA of students between high school groups. Where the SMA Village 2 (SMA-V2) group is the highest, and the lowest is SMA Village 3 (SMA-V3), based on the ANOVA test, shows that this difference is meaningful or significant (accept H1), which is presented in Figure 2.

Based on Figure 2, it shows the average and standard deviation of each data group: For region category 1 (Wonosobo Regency) it consists of C1 = 82.79 and 8.67; M1 = 76.18 and 6.52; V1 = 79.37 and 5.86. Area 2 (Boyolali Regency) it consists of C2 = 79,92 and 7,27; M2 = 81,11 and 9,87; V2 = 85,01 and 8,52. Area 3 (Temanggung Regency) it consists of C3 = 79,61 and 12,88; M3 = 83,33 and 9,74; V3 = 75,56 and 12,45. The total average is 80.34 and the standard deviation of the entire data group is 9.63.

Statistical tests show a significant influence of the Javanese Ethno-SSI Model in strengthening high school students’ EA. The model’s contribution provides an influential contribution by considering the estimated prediction parameters in the ANOVA test. ^27^

## Discussion

Based on the results of the ANOVA test ( Table 9), there was a significant influence and contribution. The contribution of the Ethno-SSI model’s influence simultaneously to increase students’ environmental awareness by 60% based on a combination of various student backgrounds based on different types of school locations and cultures. Furthermore, the regression parameter predictions show that students’ EA meets the equation Ŷ1 = 75,565 + 7,222X = 82.787. This means that the Ethno-SSI model studied can increase students’ environmental awareness simultaneously. The results of the study are reinforced by research trends over the past 10 years, which show that most science learning still separates the educational curriculum from local wisdom issues, ^3, 4, 6, 10, 28, 29^ even though social issues in society have the potential to be a source of meaningful learning. ^17, 30^ Different student backgrounds significantly influence how students understand culture and care for the environment. ^21^ However, in this study, background is not only seen from the school of origin, but also the habits and character of students in responding to surrounding traditions and culture. The importance of strengthening Environmental Awareness (EA) in high school students is based on the concrete reason that increasingly advanced technology ^31^ results in the erosion of cultural heritage rooted in society.

EA is demonstrated based on concern for, protecting, and preserving the environment so that students are not apathetic toward local wisdom as a national identity. ^32^ The shift in social behavior is increasingly evident with the role of advanced technology, enabling local wisdom education to prepare competitive graduates ^33^ who uphold tradition and culture. ^34^ A special learning strategy is needed that utilizes social issues with ethnoscience content, namely Ethno-SSI. ^11, 12, 14– 17, 35– 39^ The theme of Javanese Culture is considered the most strategic as a learning resource for Central Javanese cultural heritage, yet it is often underutilized by most schools.

Ethno-SSI, which creates an integrated environment for students’ traditional and cultural lives, has great potential to overcome the weakness of inquiry in accustoming students to mental processes and thinking skills. ^40– 42^ The lengthy adjustment process can be overcome with an ethnoscience approach. ^43– 45^ Ethnoscience examines the knowledge systems of surrounding cultures. Learning using cultural concepts as a learning resource can improve scientific knowledge. ^13, 35^

In the Ethno-SSI reconstruction of the dreadlock ritual, the topic of measurement, quantities, and units can be integrated with ethnoscience studies through the dreadlock ritual. This procession includes many activities that can be scientifically linked to the concept of measurement, such as measuring the length of dreadlocks using a ruler or measuring tape, weighing the mass of hair with a balance, and recording the time of the event using standard units such as hours or minutes. This demonstrates that measurement processes are not only relevant in the laboratory but also present in cultural activities passed down through generations.

From an ethnoscience perspective, the dreadlock ritual incorporates local knowledge related to non-standard measurements, such as determining auspicious days based on the Javanese calendar, the position of the moon, or certain natural signs. Local communities have traditionally observed natural phenomena as a guide for the ritual, fostering a close connection between culture and practical knowledge. The cultural values also teach the importance of maintaining children’s health, symbolically cleansing oneself, and respecting ancestral heritage. ^46^

The connection with EA is evident in the community’s efforts to preserve the environment during the Ruwatan ceremony. The materials used, such as flowers, young coconut leaves, and offerings, are generally naturally sourced and biodegradable, thus preventing environmental pollution. Furthermore, there is awareness of avoiding single-use plastics and ensuring that organic waste is properly managed. Thus, learning about measurement, quantities, and units not only strengthens students’ scientific skills but also instills respect for local wisdom and a responsibility to preserve the environment.

Formal education and the process of acculturation and cultural traditions inherent in society seem separate, ^47, 48^ as indigenous knowledge or culture is experiential and cannot yet be scientifically proven to relate concrete facts to their causes. ^49– 51^ Culture is a way of life that develops and is shared by a group of people and passed down from generation to generation. ^52^ This results in a lack of appreciation for the culture of one’s own region. ^14^ The importance of the ethnoscience approach in the implementation of education aims to maintain the culture of the community so that it is not lost, relevant to the objectives of science, which are oriented towards mastering knowledge, skills, values, and attitudes so that students are able to participate in the environment. ^53– 55^

The ethnoscience approach is a conceptualization of learning activities that must be close to the environment that optimally utilizes the potential of the environment, especially local culture. ^56– 58^ Teachers not only convey theory, but also transfer values taken from learning activities through 21st-century science skills profiles and more meaningful learning. ^59^ Mastery of knowledge, skills, values, and attitudes so that students are able to participate in the environment. ^60– 62^ Local culture of the community can help teachers relate the material taught to students’ real-world situations. ^53, 63, 64^ It’s not just about environmental awareness. Ethno-SSI equips students to develop scientific literacy and solve sustainable environmental problems. Strengthening environmentally-based character education through Ethno-SSI also provides a holistic learning experience that bridges theoretical knowledge with practical application, as well as fostering environmental responsibility and creative student engagement. ^65– 67^

This research provides perspectives and insights into environmental management and survival through the support of enduring traditions. The importance of this research lies in the fact that ethno-socioscientific issues differ from region to region, yet all work toward a common goal: the sustainability of ancestral culture.

### Limitation evidence

The limitations in describing science reconstruction based on the need for relevance of science materials with different indicators of students’ environmental awareness. It is essential to investigate the factors that influence students’ environmental awareness. It is necessary to study the suitability of environmental awareness determinants in more detail about the learning sources of traditions and surrounding cultures. Ethno-SSI studies are very diverse so they will determine different ways of learning reconstruction. Each study will be different depending on the researcher’s perception in explaining science reconstruction, but with these limitations, it can be used as a reference for how to develop environmental awareness of high school students for meaningful and sustainable learning in maintaining traditions and culture as a form of the existence of the golden generation of Indonesia.

## Conclusions

The Ethno-SSI Javanese culture model contributes to students’ EA attitudes through learning designs that emphasize concern for respecting, being responsive, and preserving traditions and cultures that develop in society. Differences in school background are not important factors in strengthening EA, but are determined based on the character and habits of students in learning science. Based on the data that the average environmental awareness of SMA Desa 2 Group (SMA-V2) is the highest at 85.0068, and the lowest is SMA Desa 3 (SMA-V3) at 75.5648, based on the ANOVA test, there is a meaningful or significant difference. Based on the Corrected Model, there is an influence of all independent variables (SMA Group) together on the dependent variable (Student EA), Sig. 0.000 <0.05 indicates a valid model and significant data. Intercept: The value of the change in the dependent variable (EA) without the need for the presence of the independent variable (Ethno-SSI Javanese Culture Model), meaning that without the influence of the independent variable, the dependent variable can change its value. A value of Sig. 0.000 < 0.05 indicates a significant intercept. The influence of the high school group on the value of the student’s EA measurement matrix in the model shows a significant effect. 0.004 < 0.05, indicating that the high school group has a significant effect on EA. The smaller the Model Error value, the better the model. R Squared: The multiple determination value of all independent variables with the dependent variable shows an R Squared value of 0.60, meaning that the independent variable can influence the dependent variable by 60%. Furthermore, the contribution of the model using the ANOVA equation shows a significant parameter estimate within the prediction range of Ŷ1 = 75,565 + 7,222X = 82.787.

Research recommendations highlight the importance of local government policies in preserving traditions and culture to provide a foundation and support for education. Maintaining the identity and character of the nation with appropriate policies, as well as maintaining the uniqueness and diversity of culture in Indonesia. Tradition and culture are not just performances and cultural values, but there is an increase in community environmental awareness.

## Data Availability

[Figshare]. [Results of students’ environmental awareness responses]. [ https://doi.org/10.6084/m9.figshare.30601229.v1]. ^68^ This data is the result of students’ answers to the questionnaire and environmental awareness test at each school. Data is available under the terms of the Creative Commons Attribution 4.0 International license (CC-BY 4.0). [Figshare]. [Comparassion EA Based School Area Category]. [ https://doi.org/10.6084/m9.figshare.30601304.v1]. ^69^ This data is a comparison of students’ answers to the questionnaire and environmental awareness test at each school based on regional category. This project contains data environmental awareness. Data is available under the terms of the Creative Commons Attribution 4.0 International license (CC-BY 4.0). [Figshare]. [Questionnaire and test for Environmental Awareness Profiles using Ethno-SSI Wonosobo regency]. [ https://doi.org/10.6084/m9.figshare.31072222.v1]. ^70^ [Figshare]. [Questionnaire and test for High School Student Environmental Awareness Profiles using Ethno-SSI Boyolali regency]. [ https://doi.org/10.6084/m9.figshare.30609149.v1]. ^71^ [Figshare]. [Questionnaire and test for Environmental Awareness Profiles using Ethno-SSI Temanggung regency]. [ https://doi.org/10.6084/m9.figshare.31072243.v1]. ^72^ This project contains environmental awareness instrument. Data is available under the terms of the Creative Commons Attribution 4.0 International license (CC-BY 4.0).
