# Supplementary material for: How is the Ethno-Socioscientific Issues (Ethno-SSI) Learning Model Javanese Culture Strengthen High School Students’ Environmental Awareness?: Observational Study
Source: F1000Res. 2026 May 21;15:175. Originally published 2026 Feb 3. [Version 2] doi: 10.12688/f1000research.172474.2 (PMC13202202; doi:10.12688/f1000research.172474.2)
Supplement: Peer review history

# Reviewer report: Reviewer response for version 2

**DOI:** 10.5256/f1000research.198719.r487022

**Published:** 2026-05-28

**Authors:** Rock Finale

**Affiliations:** 1 University of Applied Sciences of Rijeka, Rijeka, Croatia

The revised version of the manuscript shows a clear and constructive effort by the authors to address the comments and recommendations provided during the review process. The manuscript has improved substantially in terms of structure, clarity, and overall presentation. Several sections have been expanded and clarified, particularly the methodological description, ethical considerations, and discussion of the educational relevance of local wisdom and environmental awareness. The authors have also incorporated additional references that strengthen the theoretical background of the study.

 The topic of the article is relevant and meaningful, especially in the context of environmental education, character education, and culturally responsive learning. The integration of Javanese cultural traditions into the Ethno-SSI learning model provides an original and contextually valuable contribution to educational research. The manuscript successfully highlights the importance of preserving local cultural heritage while promoting students’ environmental awareness and social responsibility.

 One of the strengths of the article is the rich contextual explanation of the cultural practices explored in the study. The descriptions of local traditions and their connection to science learning create an interesting interdisciplinary perspective that can be useful for educators and researchers working in similar contexts. The revised discussion section also demonstrates a stronger connection between the study findings and broader educational issues related to sustainability and local wisdom-based learning.

 In general, the manuscript is now more coherent and easier to follow than in the previous version. The language has been improved, and the overall flow of the article is more consistent. Although a few minor language and stylistic issues remain, these are relatively small and could be addressed during final proofreading.

 Overall, most of the reviewers’ comments appear to have been addressed appropriately in this revised version. The manuscript demonstrates meaningful improvement and now reaches an acceptable level for indexing.

 Minor suggestions for further improvement:

1. The manuscript would benefit from an additional round of language polishing to improve clarity and readability in several sections of the discussion.
2. Some parts of the discussion remain descriptive and could be strengthened by adding slightly deeper analytical reflection on the educational implications of the findings.
3. The presentation of several tables could be simplified and standardized to improve readability for international readers.
4. The authors may consider providing a slightly clearer explanation of how the learning model could be adapted for broader educational contexts outside the studied regions.

Is the work clearly and accurately presented and does it cite the current literature?

Partly

If applicable, is the statistical analysis and its interpretation appropriate?

I cannot comment. A qualified statistician is required.

Are all the source data underlying the results available to ensure full reproducibility?

Yes

Is the study design appropriate and is the work technically sound?

Partly

Are the conclusions drawn adequately supported by the results?

Yes

Are sufficient details of methods and analysis provided to allow replication by others?

Partly

Reviewer Expertise:

Education, environmental education, environmental awareness, attitudes, and behaviour towards the environment

I confirm that I have read this submission and believe that I have an appropriate level of expertise to confirm that it is of an acceptable scientific standard.

# Reviewer report: Reviewer response for version 2

**DOI:** 10.5256/f1000research.198719.r487020

**Published:** 2026-05-25

**Authors:** Purwo Susongko

**Affiliations:** 1 Elementary Education, Universitas Negeri Semarang Fakultas Ilmu Pendidikan dan Psikologi (Ringgold ID: 563911), Semarang, Central Java, Indonesia

OK, all my suggestions have been implemented well

 Purwio Susongko 

 Universitas Negeri Semarang

Is the work clearly and accurately presented and does it cite the current literature?

Partly

If applicable, is the statistical analysis and its interpretation appropriate?

I cannot comment. A qualified statistician is required.

Are all the source data underlying the results available to ensure full reproducibility?

Yes

Is the study design appropriate and is the work technically sound?

Partly

Are the conclusions drawn adequately supported by the results?

Yes

Are sufficient details of methods and analysis provided to allow replication by others?

Partly

Reviewer Expertise:

Educational assesment and Science Education

I confirm that I have read this submission and believe that I have an appropriate level of expertise to confirm that it is of an acceptable scientific standard.

# Reviewer report: Reviewer response for version 1

**DOI:** 10.5256/f1000research.190203.r457731

**Published:** 2026-03-04

**Authors:** Purwo Susongko

**Affiliations:** 1 Elementary Education, Universitas Negeri Semarang Fakultas Ilmu Pendidikan dan Psikologi (Ringgold ID: 563911), Semarang, Central Java, Indonesia

**Brief Summary of the Article**

 This study investigates the effectiveness and contribution of the Ethno-Socioscientific Issues (Ethno-SSI) learning model based on Javanese culture in strengthening high school students’ Environmental Awareness (EA) in Central Java, Indonesia. The authors employed a Design-Based Research (DBR) framework combined with quantitative (ANOVA) and qualitative approaches across nine high schools. The findings indicate statistically significant differences in EA across school groups, with the model reported to contribute up to 60% (R² = 0.60) to EA outcomes.

 The article addresses a relevant topic in environmental education by integrating local wisdom and socioscientific issues into formal learning contexts.

**Major Comments**

 1. Alignment Between DBR Framework and Statistical Analysis

 While the manuscript states that it uses a Design-Based Research (DBR) approach, the empirical analysis primarily reflects cross-sectional group comparisons using one-way ANOVA. DBR typically involves iterative cycles of design, implementation, evaluation, and refinement. However, the analytical section does not clearly demonstrate these iterative phases nor explain how the statistical findings inform model refinement.

 The authors should:

- Clarify how each DBR phase was operationalized.
- Explain how findings from implementation cycles informed subsequent refinement.
- Strengthen coherence between the DBR conceptual framework and the statistical procedures.

2. Causal Language and Interpretation of Results

 The manuscript frequently implies that the Ethno-SSI model *causes* improvement in environmental awareness. However, the statistical design (group comparison using ANOVA without clear randomized controls or longitudinal tracking) primarily supports associative relationships rather than causal inference.

 The authors should:

- Avoid causal language unless supported by stronger experimental control.
- Reframe conclusions to reflect associative findings where appropriate.
- Clarify whether pre-test/post-test comparisons were conducted and how they were analyzed.

3. Statistical Reporting and Interpretation

 Although ANOVA is appropriately used for comparing group means, several interpretative elements require clarification:

- The reported R² values appear inconsistently described (e.g., 0.91 vs 0.60 adjusted R² in the table). This should be clarified.
- Effect sizes should be explicitly reported and interpreted.
- The assumptions of ANOVA (normality and homogeneity) are mentioned, but violations in some groups (e.g., normality tests in certain schools) should be addressed more transparently.

A more rigorous statistical interpretation would strengthen the scientific validity of the findings.

 4. Instrument Development and Validation

 The manuscript mentions Fischer’s Environmental Awareness framework and provides links to instruments via Figshare. However, the following details should be elaborated:

- Reliability indices (e.g., Cronbach’s alpha).
- Construct validity procedures.
- Factor structure (if applicable).
- Scoring procedures for the essay-based EA test.

More detailed reporting would significantly improve replicability.

 5. Literature Balance and Theoretical Framing

 The manuscript relies heavily on self-citations (approximately 30% of references). While relevant, a broader engagement with international literature on:

- Environmental awareness,
- Character education,
- Nature of Science (NOS),
- Scientific literacy,

would strengthen the theoretical grounding.

 In particular, I recommend connecting Environmental Awareness (EA) and Character Education with the Nature of Science framework. For example:

 Susongko, P., Wahab, N. B. A., Arfiani, Y., & Kusuma, M. (2024). *Validation and Implementation of 3-Dimensional Scientific Literacy Test (Lisa3D Test): Measuring Scientific Literacy for Senior High School Students based on Scientific Reasoning, Scientific Inquiry, and Nature of Science.* Jurnal Pendidikan IPA Indonesia, 13(3).

 Integrating the Nature of Science dimension would provide stronger conceptual grounding for how Ethno-SSI supports not only awareness but scientific literacy development.

**Minor Comments**

1. Some tables require clearer labeling and more concise explanation in the text.
2. The abstract could be slightly refined to clarify the study design and avoid overstatement of effectiveness.
3. Language editing would improve readability in several sections.
4. Ethical approval and consent details are extensive; they may be condensed for clarity

**Strengths of the Article**

- Addresses a timely and relevant issue in environmental education.
- Integrates cultural heritage and socioscientific issues in an innovative way.
- Provides openly accessible datasets (strong transparency and reproducibility).
- Offers potential implications for culturally responsive environmental education policy.

**Overall Recommendation**

 Based on the above considerations, I maintain the status:

**Approved with Reservations**

 The article is relevant and potentially valuable, but revisions are needed to strengthen methodological clarity, statistical interpretation, and theoretical framing before it can be considered fully scientifically robust.

Is the work clearly and accurately presented and does it cite the current literature?

Partly

If applicable, is the statistical analysis and its interpretation appropriate?

I cannot comment. A qualified statistician is required.

Are all the source data underlying the results available to ensure full reproducibility?

Yes

Is the study design appropriate and is the work technically sound?

Partly

Are the conclusions drawn adequately supported by the results?

Yes

Are sufficient details of methods and analysis provided to allow replication by others?

Partly

Reviewer Expertise:

Esducational assesment and Science Education

I confirm that I have read this submission and believe that I have an appropriate level of expertise to confirm that it is of an acceptable scientific standard, however I have significant reservations, as outlined above.

# Reviewer report: Reviewer response for version 1

**DOI:** 10.5256/f1000research.190203.r457730

**Published:** 2026-02-26

**Authors:** Ika Lestari

**Affiliations:** 1 Faculty of Education, Universitas Negeri Jakarta (Ringgold ID: 95367), East Jakarta, Special Capital Region of Jakarta, Indonesia

1. In the background section, references are still found in the author's native language (Indonesian). The intended journal is a reputable international journal, and its readers will come from various countries. Therefore, it is recommended that references in the researcher's native language not be used in this manuscript.
2. The research question regarding the Javanese Cultural Ethno-Sociological Issues Learning Model (Ethno-SSI) to Strengthen High School Students' Environmental Awareness has not been reflected in the results and discussions, even though that is what was produced in the DBR.
3. What are the characteristics of the final design for the model? This research appears more experimental than DBR.
4. In the abstract, it is stated that a qualitative approach is used, but the method does not yet describe the data collection instruments, the thematic analysis, or the triangulation.
5. The results of expert validation have not been explained. DBR emphasizes the process of model improvement through formative testing, so expert validation data is needed to produce an initial prototype.

Is the work clearly and accurately presented and does it cite the current literature?

Partly

If applicable, is the statistical analysis and its interpretation appropriate?

Yes

Are all the source data underlying the results available to ensure full reproducibility?

Partly

Is the study design appropriate and is the work technically sound?

Partly

Are the conclusions drawn adequately supported by the results?

Partly

Are sufficient details of methods and analysis provided to allow replication by others?

Partly

Reviewer Expertise:

Educational Technology, Elementary School Teacher Education, and Instructional Media

I confirm that I have read this submission and believe that I have an appropriate level of expertise to confirm that it is of an acceptable scientific standard, however I have significant reservations, as outlined above.

# Reviewer report: Reviewer response for version 1

**DOI:** 10.5256/f1000research.190203.r457722

**Published:** 2026-02-23

**Authors:** Rock Finale

**Affiliations:** 1 University of Applied Sciences of Rijeka, Rijeka, Croatia

Dear Sir/Madam, I am presenting the comments in the same order as the questions. In addition, I also provide a general impression of the article at the end.

 1. There are some problems with the references, as one author (Khoiri, A.) is cited 21 times out of 69, which represents 30% of all references (he/she is also the author of this article). Notwithstanding the above observation, the manuscript is understandable and logically organized, but it is not always methodologically rigorous in linking the research design to the statistical inference. The manuscript would benefit from clearer alignment between the methodology and the DBR analysis, better interpretation of the statistical results, and better interpretation of the tables. 

 2. The study design is conceptually relevant. Statistical analyses are generally adequate for comparing group means, but the technical interpretation of the results, especially regarding the influence of the model and the effects of the intervention, could be more in-depth. The manuscript, in my opinion, is only partially methodologically adequate, as it is based primarily on linking the Ethno-SSI learning model to observed environmental awareness outcomes. The actual implementation of the research design raises concerns about its consistency with the DBR methodology - I think the DBR model is well illustrated in Figure 1.

 This study relies primarily on cross-sectional testing of environmental awareness (EA), questionnaires, and one-way ANOVA comparisons between school groups.

 3. Although the manuscript describes the general methodological framework and statistical procedures, it does not provide sufficient procedural or analytical detail to allow other researchers to fully replicate the study. Greater transparency regarding the implementation of interventions, instrument development, and data analysis would be needed to support replicability. The procedures, scale structure, validation methods, and reliability indices are only briefly mentioned.

 4. The analysis supports the identification of links between school context and EA outcomes but does not fully justify conclusions about the effectiveness of the Ethno-SSI learning model.

 I kindly recommend that the statistical analyses and interpretation be reviewed by a qualified statistician.

 5. The manuscript provides access to the underlying source data through publicly available Figshare repositories. These include anonymized questionnaire responses, environmental awareness test results, and the instruments used for data collection. All datasets are shared under a Creative Commons Attribution 4.0 International (CC-BY 4.0) license, allowing independent verification and reanalysis. Therefore, the availability of source data supports the reproducibility and transparency of the reported findings.

 6. Although the statistical findings support the existence of differences in environmental awareness across school contexts, the analyses conducted do not fully support conclusions about the effectiveness of the Ethno-SSI learning model. A more in-depth interpretation would indicate a stronger associative nature of the findings and avoid attributing causality to the intervention without additional controls.

 7. The abstract is modestly written. If this is the magazine's instruction, I agree with the length and style of writing.

 8. A consent form is acceptable to the editor and/or author, but does not necessarily form part of the article, or can be shortened.

 Overall, the manuscript addresses a relevant and current topic by exploring the integration of culturally informed social science approaches to improving students’ environmental awareness. The study is logically structured, and the use of quantitative analysis provides an initial empirical basis for examining differences in outcomes across educational contexts. There is a need to strengthen the coherence between the aforementioned DBR framework and the analytical procedures, as the current analysis primarily reflects comparisons of groups rather than the phased cycles of the design implementation process that characterizes DBR. The interpretation of statistical results should be clearer to avoid implicating the causal influence of the Ethno-SSI model, where only associative relationships are tested.

 With revisions that address methodological and interpretative issues, the manuscript could offer a more concrete contribution to the field of culturally responsive environmental education.

 If appropriate, given the content of the manuscript, I am adding two DOIs that the author may include in the manuscript and add to the references.

Is the work clearly and accurately presented and does it cite the current literature?

Partly

If applicable, is the statistical analysis and its interpretation appropriate?

I cannot comment. A qualified statistician is required.

Are all the source data underlying the results available to ensure full reproducibility?

Yes

Is the study design appropriate and is the work technically sound?

Partly

Are the conclusions drawn adequately supported by the results?

Yes

Are sufficient details of methods and analysis provided to allow replication by others?

Partly

Reviewer Expertise:

Education, environmental education, environmental awareness, attitudes, and behaviour towards the environment

I confirm that I have read this submission and believe that I have an appropriate level of expertise to confirm that it is of an acceptable scientific standard, however I have significant reservations, as outlined above.

## References

[1] : Can We Raise the Level of Environmental Awareness Through Art?. *Social Education Research* .2025; 10.37256/ser.6120255368 147-163 10.37256/ser.6120255368

[2] : ECTA Approach to Teaching Environmental Content.2026; 10.1007/978-3-032-05760-0_3 47-69 10.1007/978-3-032-05760-0_3
